# Exploration of Alcohol Consumption Behaviours and Health-Related Influencing Factors of Young Adults in the UK

**DOI:** 10.3390/ijerph17176282

**Published:** 2020-08-28

**Authors:** Sunbal Naureen Bhatti, Lampson M. Fan, Adam Collins, Jian-Mei Li

**Affiliations:** 1 School of Biological Sciences, University of Reading, Reading RG6 6AS, UK; jian-mei.li@reading.ac.uk; 2 Department of Cardiology, The Royal Wolverhampton NHS Trust, Wolverhampton WV10 0QP, UK; lampson.fan1@nhs.net; 3 Faculty of Health and Medical Sciences, University of Surrey, Guildford, Surrey GU2 7YH, UK; a.collins@surrey.ac.uk

**Keywords:** alcohol consumption, ethnicity, physical activity, screen-time, sedentariness, smoking, university students, young adults

## Abstract

Hazardous alcohol consumption is ranked above illicit drug use with regards to health deterioration and social and economic burden. This study sought to clarify the factors influencing alcohol consumption and its prevalence in young adults. Demographics, alcohol consumption and lifestyle information were gathered via anonymous questionnaires during 2011–2019, crossing Reading, Surrey and Farnborough universities, UK. Controlling for confounders, a multinomial logistic regression was performed using SAS^®^ 9.4 software. A total of 1440 students (43.5% males, 56.5% females; 54.4% Caucasians) with a mean (SD) age of 19.9 (2.73) were included. Among them, 68.9% consumed alcohol frequently and 31.7% had ≥12 units/week. Statistical analysis revealed that males consumed twice more alcohol than females, odds ratio (OR) 1.67 (95% confidence interval (CI) = 1.34–2.09), *p*-value < 0.01. Caucasians consumed up to five times more alcohol than other ethnicities, OR 4.55 (3.57–5.56), *p*-value < 0.01. Smokers consumed three times more alcohol than non-smokers, OR 2.69 (1.82, 3.99), *p*-value < 0.01. In general, the levels of alcohol consumption were positively associated with the levels of physical activity, OR 2.00 (1.17–3.42), *p*-value < 0.05 and negatively associated with recreational sedentary screen-time activities in males, OR 0.31 (0.12–0.86), *p*-value = 0.03. Focusing alcohol interventions toward Caucasians, smokers and physically active students, particularly males, may guide university strategies to reduce alcohol-related societal harm and risks of morbidity and mortality.

## 1. Introduction

Young adulthood (18–25 years) is a period of exploration, excitation, exposure to peer challenge and adaptation of lifestyle behaviours that cement during later adulthood [1]. University is a unique environment where young adult males and females of different ethnic cultural backgrounds gather together. Here they are unbound by familial constraints with the freedom to make lifestyle choices to suit personal needs. This university experience involves a variety of small-scale environments, termed micro-environments, including both physical and social settings which contribute to the establishment of behavioural patterns [2]. Social norms in micro-environments encouraging alcohol intake, such as bars/clubs, student halls and campus events increase the risk of unsafe alcohol consumption greatly at university [3,4]. This manifests predominately by integrating into binge drinking (≥5 drinks on one occasion) as part of university cultures [5]. Although the UK government recommended alcohol limit is <14 units/week (≈6 pints/week) for both male and female adults [6], exceeding the limit has been identified as a problematic behaviour among university students [3].

Tiered above all other illicit narcotics [7], harmful alcohol intake is defined as a level of drinking that leads to physical or cognitive harm to oneself or others namely, families and society [8]. The self-inflicted damage arising from excessive alcohol consumption is a prevalent cause of premature mortality due to liver failure and a risk factor for many other diseases [9]. It is also recognised that neurotoxic effects mediated by an excessive level of alcohol exposure at young ages increases the risk for brain injury [10] and reductions of brain volume in the subcortical and temporal regions, which lead to poorer academic performance, depression and antisocial behaviours [11,12]. Albeit there is evidence to suggest that low levels of alcohol consumption have protective effects against cardiovascular diseases [13], periodic binge drinking at young ages can precipitate various forms of morbidity and/or alcohol dependence in later adulthood [1]. Therefore, identifying the factors that influence the level of alcohol consumption at university is important for developing improved strategies to protect this young generation.

Previous studies had highlighted that demographic, psychological and behavioural factors had an important role in the levels of alcohol consumption by university students [14,15]. However, there were discrepancies in the literature. For example, some studies reported the male gender as a determinant of greater alcohol intake [15], whereas others describe similar levels of alcohol consumed across genders [16]. Increases in alcohol consumption among young adult females [17] are worrying and imperative to explore as females incur enhanced susceptibility to the harmful effects of alcohol than males upon drinking equal amounts [18]. Caucasians are reported to consume greater levels of drinking compared to minority ethnic groups [19]. Reports also highlight an increase in heavy episodic drinking in Asian students to match Caucasians [20]. Physical activity (PA) had been described to reduce the association between alcohol consumption and risk of death [21] whereas others had found an association between higher PA and high levels of alcohol consumption [22]. Another significant lifestyle factor was the amount of recreational sedentary screen-based activities, i.e., internet use and watching television, which was found to be associated with alcohol use [23]. The relation between high levels of alcohol consumption and recreational sedentary screen-time behaviour remains controversial with reports failing to observe any association between screen-based activities and alcohol [24], while others have documented a positive association [25].

Almost half of young adults in the UK consume alcohol with many engaging in hazardous drinking behaviours [26]. Interventions to control this misuse require attention as alcohol use disorders are placing enhanced burden upon the National Health Service and causing increased alcohol-specific deaths in England relative to figures from the past decade [27]. As certain groups are more susceptible to alcohol marketing and developing alcohol dependence than others, the targeting of vulnerable groups to reduce risk has been advocated [28]. In consideration of the controversial evidence for drinking behaviours in groups of young adults, the identification of certain groups and behaviours that influence alcohol consumption may pose challenge in the attempts to reduce alcohol-related harm in university students. Given that alcohol dependence is postulated to initialise from drinking behaviours during young adulthood [1], shedding light on which groups are most vulnerable may better inform public health policy to tailor alcohol-related interventions toward high risk groups.

Herein, we hypothesise that alcohol consumption is prevalent among UK university students and that certain sub-groups of individuals exhibiting specific health-related behaviours are at greater risk of higher quantities of alcohol intake. The aims were to elucidate the occurrence of alcohol consumption quantities across genders and the association between this intake and certain demographics and health-related lifestyle factors. By gathering and analysing data from 1440 students crossing three different universities in the UK (Reading, Surrey and Farnborough) this study endeavoured to disentangle the controversies of factors that influence the levels of alcohol consumption in young adults at university. Given the fact that alcohol intake is a modifiable factor, ascertaining the determinants of alcohol consumption is valuable for developing contemporary alcohol-awareness initiatives to protect those who are at risk of developing alcohol-related diseases across the myriad of young adults in the UK.

## 2. Methods

### 2.1. Ethical Consent

The study had obtained the approvals from the Research Ethics Committees of the School of Biological Sciences, University of Reading, UK (17-18-25); the Ethics Committee of the Faculty of Health and Medical Sciences, University of Surrey, UK (EC/2011/38); and the Faculty Ethics Sub-Committee, University Centre Farnborough (UCF-11-05-18), UK in accordance with their institutional procedures. 

### 2.2. Study Criteria, Recruitment and Data Collection

Volunteers, both male and female, were randomly recruited from the 1st year and 2nd year undergraduate population at the University of Reading (Reading, UK), University of Surrey (Guildford, UK) and University Centre Farnborough (Farnborough, UK) between the academic years of 2011–2019. Recruitment was achieved by probabilistic sampling using the three-university student census as a sampling frame and sampling randomly within departmental strata and then sampling randomly within lectures. Since the study intended to obtain a representative sample of UK university students, each university selected reflected percentages of gender, domicile and ethnicity aligning with those reported for the UK population by Universities UK [29,30,31,32]. Inclusive criteria were undergraduate students aged between 18 and 35 years, within two years of university life and without declared medical conditions. Those aged above 35 years, surveys with missing data and participants reporting debilitating or medical conditions which would otherwise affect ability to partake in PA (i.e., bone/joint disorders) were excluded from the study.

### 2.3. Study Design

Preceding the dissemination of surveys, participants were provided with study information and consent forms. Data were gathered from volunteers via self-completed, hard copy and anonymous questionnaires, which were conducted in a multiple-choice format with opportunities for constant sum and open-ended responses to address lifestyle components. The questionnaire included: (1) demographic data encompassing gender, age, anthropometric information, ethnicity, medical conditions and home country of residence; (2) quantity and frequency of alcohol consumption which were categorised into the groups 0, <3, 3–11, and ≥12 units/week to generate categories of non-drinker (including pre-drinkers), lowest-, average- and highest-consumers of alcohol, respectively, within the sample of students who may not necessarily exceed the government recommended alcohol limit but are beginning to develop poor alcohol habits at this stage of young adulthood. Quantities of alcohol (i.e, spirits/beer) were calculated using the formula: strength (alcohol by volume measured as a percentage of alcohol) × volume (mL) ÷ 1000 = units of alcohol; (3) smoking quantity and frequency (cigarettes/week), which were categorised into non-smokers (including pre-smokers) and smokers; (4) levels and duration of PA, which was categorised in consideration of the UK government PA Guidelines [33] and further grouped into non-active, active below PA guidelines, active above PA guidelines and very active (0, <2.5, 2.5–8, >8 h/week, respectively) and gym attendance (attend or do not attend); (5) sleep duration, which was categorised in accordance with the recommended hours for young adults (<7 and ≥7 h/day) [34]; (6) recreational sedentary screen-time behaviours including computer use, watching TV and video gaming, which were grouped into <4 and ≥4 h/day consistent with a previous paper reporting increased risk for morbidity in individuals occupying the latter group [35] and; (7) self-perceptions of physical fitness (fit or unfit). Body Mass Index (BMI) was calculated and the participants were characterised according to their bodyweight status by separation into three categories predefined by the National Institutes of Health [36] as follows: underweight (BMI < 18.5 kg/m^2^), normal weight (BMI 18.5–24.9 kg/m^2^) and overweight/obese (BMI ≥ 25/≥ 30 kg/m^2^).

### 2.4. Statistical Analysis

Demographic data were analysed and comparisons of anthropometric information were achieved using descriptive statistics, reported as mean and standard deviation (SD), *t*-tests or chi-square tests. The statistical analysis was performed using SAS software, Version 9.4 (SAS Institute Inc., Cary, NC, USA). Statistical analysis of alcohol consumption was performed using a multinomial logistic regression with the genmod procedure. The ordinal response variable of alcohol consumption (0, <3, 3–11, and ≥12 units/week) was examined for associations with the predictors: gender, BMI, ethnicity, smoking status, PA, sleep duration, recreational sedentary screen-time and perception of fitness, which yielded adjusted odds ratios (OR) as effect measures. Statistical significance was accepted at 5% using a Wald chi-square test [37]. The point estimate will be presented followed by 95% confidence intervals for each explanatory variable in brackets.

## 3. Results

### 3.1. Sample Demographics and Lifestyle Behaviours

A cohort of 1530 students were recruited and among them 1440 satisfactory responses were included in the study. The response rate (94.1%) was obtained by dividing the number of complete survey data provided by the entire number of eligible entries in the sample. Demographic information is provided in Table 1. There were 627 (43.5%) males and 813 (56.5%) females with a mean (SD) age of 19.9 (2.73) of which a large proportion (783, 54.4%) were Caucasian and many lived in Europe (85.4%). The average BMI was 21.8 kg/m^2^ across the cohort with 264 (18.3%) students classified as overweight/obese and 103 (7.2%) students classified as underweight.

Of those who were current smokers (150, 10.4%), there were 66 (44.0%) students who smoked ≥7 cigarettes/week, a pre-defined cut-off evidenced to increase the risk for CVDs [38]. Analysis of self-assessed PA levels revealed that 198 students (13.8%) failed to engage in any form of PA and 513 (35.6%) students engaged in <2.5 h/week PA, falling below UK government recommendations [33]. A considerable proportion of students (623, 43.3%) reported to perform 2.5–8 h/week of PA, while 106 (7.4%) engaged in >8 h/week of PA wherein there were more males (10.0%) than females (5.3%), *p* < 0.01. A worrying proportion of students (53.7%) are not abiding by the sleep recommendations (≥7 h/day) [34]. There were 859 students (59.7%) who expended ≥4 h/day on recreational sedentary screen-time. With regards to fitness perceptions, there were 782 (54.3%) students who perceived themselves to be unfit with the number of females greater than that of males, *p* < 0.01.

### 3.2. Alcohol Consumption

**Figure 1:**
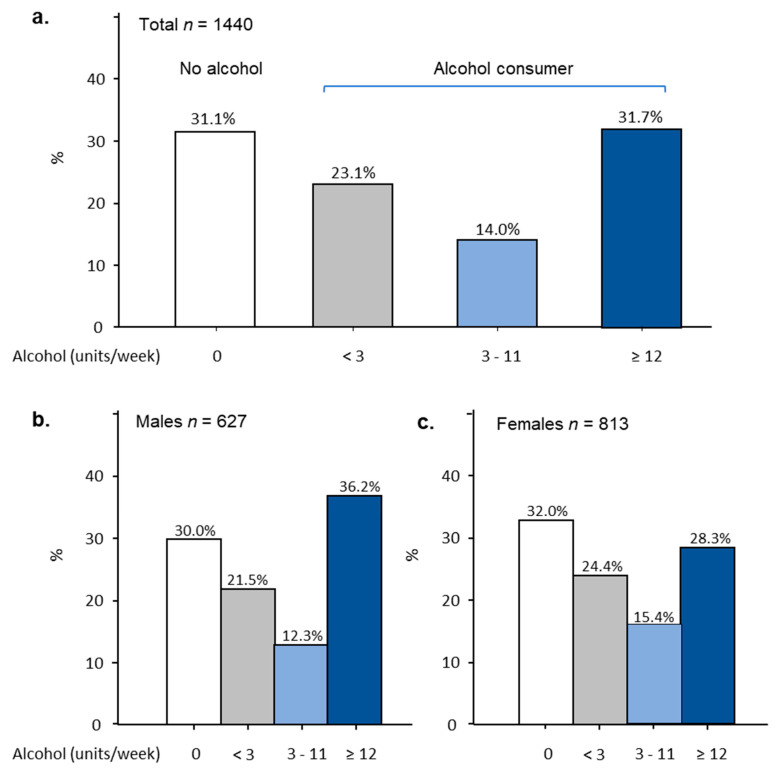
Levels of alcohol consumption (units/week) and distributions in university students. Distribution of (**a**) total (*n* = 1440); (**b**) male (*n* = 627); (**c**) female participants (*n* = 813) separated according to the levels of alcohol consumption.

A large proportion of students, 992 (68.9%), reported to drink alcohol and the remainder (448, 31.1%) were abstinent from alcohol. Figure 1 provides detailed information on the quantity (units/week) of alcohol consumed. We found that 31.7% of participants (457) consumed ≥12 units/week with male values exceeding that of female values by 7.9%, OR 1.67 (1.34–2.09), *p*-value < 0.01. Around 14% of participants (202) consumed 3–11 units/week and 23.1% of participants (333) consumed <3 units/week where there were more females than males in both categories. The levels of alcohol consumption for students exhibited a bimodal pattern where either very little or close to the limit of <14 units/week was consumed.

### 3.3. The Factors Correlated with Alcohol Consumption

**Figure 2:**
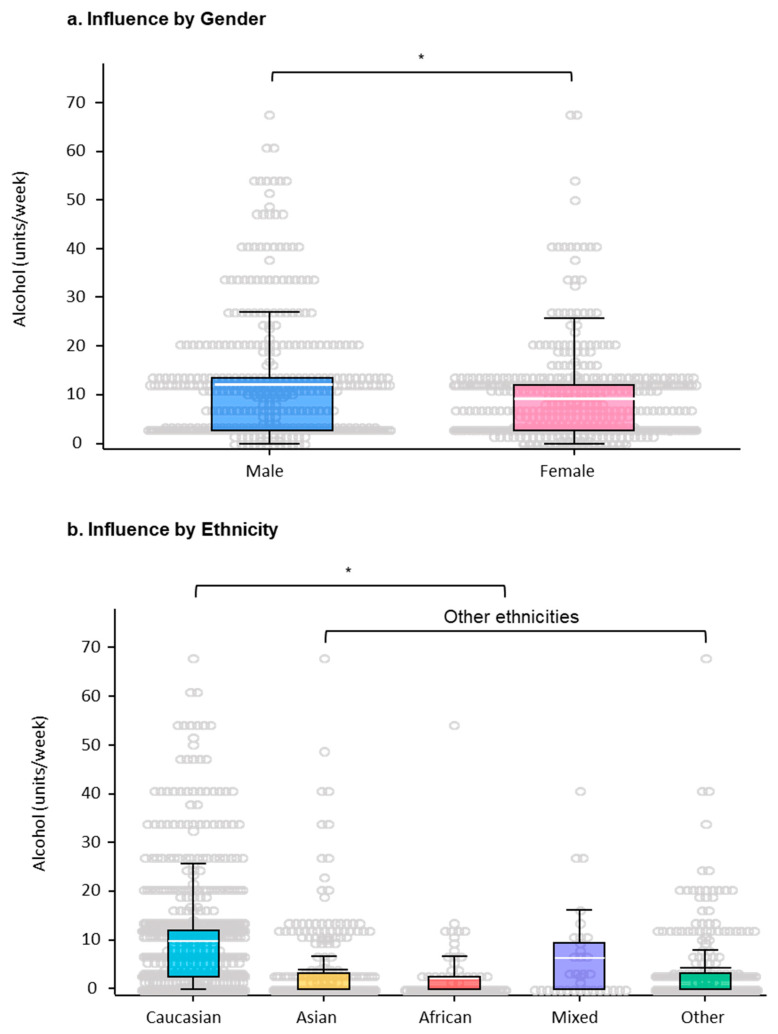
Demographic factors that influence the levels of alcohol consumption (units/week) by university students. Scatter boxplots representing (**a**) gender and (**b**) ethnicity and the levels of alcohol consumption (units/week, *y*-axis); (**a**) * odds ratio (OR) 1.67 (1.34–2.09), *p*-value < 0.01, between females versus males and (**b**) * OR 4.55 (3.57–5.56), *p*-value < 0.01, between the values of other ethnicities versus Caucasian ethnicity. Solid white/black bars in the boxes represent means.

Gender: Statistical analysis revealed that gender was an influential factor of the levels of alcohol consumed. More males (36.2%) than females (28.3%) were in the category of ≥12 units/week, OR 1.67 (1.34–2.09), *p*-value < 0.01. Similar proportions of both males and females were in the categories of non-drinker or below 11 units/week (Figure 2a).

Ethnicity: Another significant factor emerging from the statistical model was cultural background or ethnicity across genders (Figure 2b, Table 2). Among the 783 Caucasian participants, 670 (85.6%) drank alcohol, whereas among the 657 participants of other ethnic backgrounds, only 332 (50.5%) drank alcohol. For those who drank ≥12 units/week, there were 342 (43.7%) Caucasians, which was almost twice the number of non-Caucasians (115, 17.5%). The multinomial regression analysis showed that Caucasian ethnicity arose as a significant factor associated with increased alcohol consumption for both males and females, OR 4.55 (3.57–5.56), *p*-value < 0.01. Further analyses of the levels of alcohol consumption and ethnicities by gender are tabulated in Supplemental Table S1.

**Figure 3:**
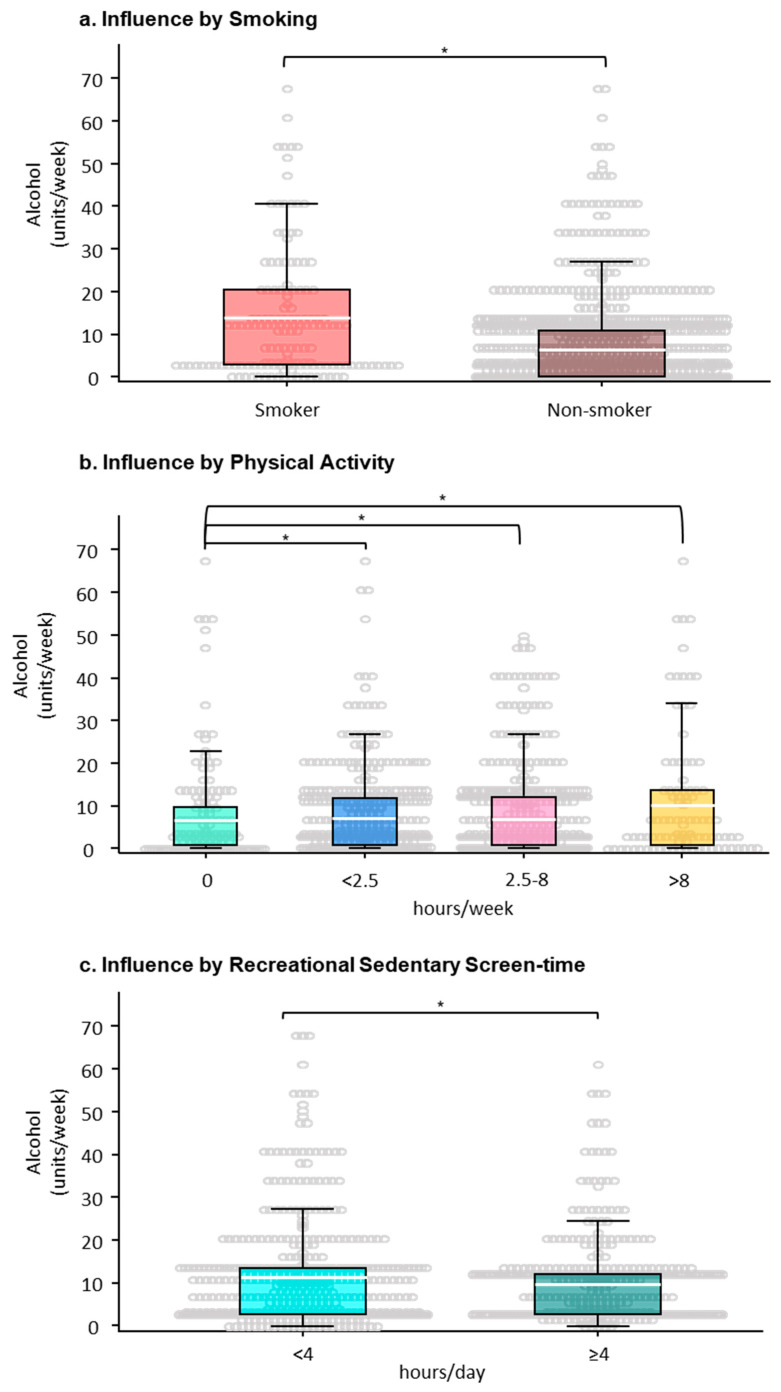
Modifiable factors that influence the levels of alcohol consumption (units/week) by university students. Scatter boxplots representing (**a**) smoking, (**b**) physical activity levels (hours/week), and (**c**) levels of recreational sedentary screen-time (hours/day) and the levels of alcohol consumption (units/week, *y*-axis); (**a**) * OR 2.69 (1.82, 3.99), *p*-value < 0.01, between non-smoker versus smoker; (**b**) * OR 2.00 (1.17–3.42), *p*-value < 0.05, between physically inactive students (0 h/week) versus the values of varying levels of physical activity; (**c**) * OR 0.74 (0.58–0.94), *p*-value < 0.02, between low (<4 h/day) versus high (≥4 h/day) recreational sedentary screen-time. Solid white bars in the boxes represent means.

Smoking: Although only 150 (10.4%) participants in this study declared themselves as current smokers either socially or regularly (Table 1), adjusted for other confounding variables, smoking emerged as a significant variable correlating with greater quantities of alcohol consumption across genders, OR 2.69 (1.82, 3.99), *p*-value < 0.01 (Figure 3a, Table 2).

Physical activity: The levels of PA was another variable associated with quantity of alcohol consumption, particularly in males (Figure 3b, Table 2). Although there were only 7.4% of students (106) who reported to take part in >8 h/week of PA, the amount of alcohol intake in this group (10.00 (14.76) units/week) was higher than others (Figure 3b), which was significantly higher in males, OR 5.47 (2.12–10.11), *p*-value < 0.01. Students who engaged in >2.5 h/week of moderate exercise or PA, consumed more alcohol (7.08 (9.97) units/week) than those who did not engage in PA (6.62 (11.87) units/week)) (Figure 3b), which revealed significance for males, OR 3.76 (1.71–8.28), *p*-value < 0.01. Even for students who reported PA levels below the UK guidelines (<2.5 h/week) they persisted to have significantly more alcohol intake (7.10 (10.89) units/week) than those who did not engage in PA, OR 1.63 (1.13–2.36), *p*-value < 0.01. It was also noticed that non-alcohol consumers were within the group who did not participate in any form of exercise.

Recreational sedentary screen-time: We also found recreational sedentary screen-time engagement (sedentariness) to be a factor negatively correlated with the levels of alcohol consumption in males, OR 0.31 (0.12–0.86), *p*-value = 0.03 (Table 2). Those students who expended ≥4 h/day on recreational screen-based sedentary activities (859, 59.7%) consumed less alcohol than others (Figure 3c). No correlation was found between the levels of alcohol consumption and BMI, sleep duration or fitness perceptions.

## 4. Discussion

Hazardous alcohol consumption is ranked above illicit drug use causing harm to our society, economics and health [9]. Excessive alcohol intake is frequently associated with unhealthy and risk-taking behaviours including poor dietary behaviours, false self-perceptions and psychological and anti-social disorders [39,40,41]. This study sheds light on gender, ethnicity, smoking, levels of PA or recreational sedentary screen-time as influential factors in the levels of alcohol consumption examined from information gathered from 1440 university students over nine years and covering three UK universities. A bimodal fashion of drinking was apparent, with the highest levels of alcohol intake (≥12 units/week) encompassing the most prevalent group among students, particularly males, closely followed by those who were abstinent. Compared to a study in 2016 where 90% of students reported to consuming some form of alcohol, herein a smaller, yet noteworthy, percentage (68.9%) reported to drink alcohol. It is documented that 25% of university students participate in intentional binge drinking once a week or more [42]. Young adults at university are particularly vulnerable to risk-taking behaviours as they seek social reward by mirroring substance-using peers while in the absence of parental control [43], posing substance-related harm to health. Herein, factors which encourage such risk of alcohol-related harm are addressed to clarify discrepancies in this field and may support the development of contemporary, gender-specific risk-reducing university programmes.

We found that male students were more engaged in excessive alcohol intake (≥12 units/week) in comparison to their female peers. This is supported by a previous UK study reporting that the male gender is positively associated with alcohol consumption and they more easily engage in hazardous alcohol consumption at university social engagements [15]. Having a Caucasian background was reported to enhance risk for harmful alcohol consumption compared to other ethnicities [19,44]. Similarly, we found that on average, Caucasian students consumed a greater level of alcohol than those from other ethnic backgrounds. Although the pattern of drinking varies between ethnic groups, culture, background and societal influences were thought to play an important role [44]. As part of the drinking culture in Western countries, drinking behaviour is accepted as a group conduct and a social norm among Caucasians [44], which is mirrored at university wherein young adults are gathered and free to express their personal autonomy to drink alcohol. The synergistic and additive consequences of smoking and drinking pose strong risk for cardiovascular disease (CVDs), cancers and premature mortality [45]. Although there were a relatively small number (150) of student smokers in our study, we found that smoking correlated positively with the levels of alcohol consumption. Our discovery parallels the reciprocal association between alcohol and tobacco use reported by international colleagues [46], and in the UK, where there is increased likelihood of students reporting smoking if they also reported high levels of alcohol intake [3]. The risk-taking culture of university young adults and the long-term health damage due to combined alcohol and smoking provides indication for the need to regulate these activities at universities.

One important finding from our study is that the levels of PA positively influenced the levels of alcohol consumption such that students who spent more time in PA consumed more alcohol, particularly in males. Previously, heavy drinking behaviours were found in those students who participated in sports (social and team-based) [47], whereas a more recent meta-analysis reports that abiding by the PA recommendations reduces the association between alcohol consumption and risk of death [21]. The incongruence with the observation herein may be ascribed to the attraction toward heavy drinking cultures in university-based sports, teams and various clubs during their celebratory or social time. Worryingly, an alcoholic beverage often contains more calories than other forms of social drink, as alcohol contains 7 calories per gram whereas carbohydrates contain only 4 calories per gram. Exercise helps to reduce calories and avoid weight gain after excessive drinking. A longitudinal study shows that higher intensities of PA offers protection from the risk of fatty liver [48]. However, we had reported previously that starting university life did not lead to the adoption of a healthy lifestyle [4]. As there is no system in place to limit or record the sale of alcohol (per occasion) provided to students in university bars, once a binge drink starts it may to be difficult for students to refuse more alcohol among the peer and social influence thus alcohol and PA awareness campaigns for these at-risk groups may be worthwhile.

Another novel discovery from our study is that the levels of recreational sedentary screen-time in males is negatively and significantly associated with levels of alcohol consumption. We found that students who spent more time on recreational screen activities drank less alcohol, which contrasts with others describing a positive association [23]. A potential explanation may in part follow the displacement hypothesis wherein more attention is given to recreational screen-based activities and less interest (or less time) for social binge drinking. Although a sedentary lifestyle increases the risk of cardiovascular and metabolic diseases, a balance between recreational screen-time, PA duration and levels of alcohol consumption is important for the wellbeing of university students.

Being overweight/obese is a prevailing public health concern in young people and strongly associated with risk for CVDs. Research describes the energy derived from alcohol intake as a risk factor for obesity [49]. In our study, there were only 264 students (18.3%) classified as overweight/obese, and BMI was not correlated with alcohol consumption which may be due to students: (1) not drinking over the limit and (2) becoming new alcohol consumers, the weight gain effects of which are observed in later years. In line with this, studies show that only excessive consumption of alcohol was associated with weight gain in young adults [49] yet low levels of drinking failed to reflect correlation with any gain in adiposity [50]. A previous study had found a synergistic link in young adults between sleep quality, alcohol consumption and academic performance [51]. Conversely, we did not find any association between self-reported sleep duration and the levels of alcohol consumption. The patterns of sleep is also an important measure for optimal health and well-being. Further study is required to clarify the association between sleep and alcohol consumption.

The Medical Research Council describes formative research as central in targeting appropriate groups for effective strategies for behaviour change [52]. In their research, Epton et al. had developed an intervention to convey health messages to university student groups found to be at higher risk of binge drinking by targeting factors which were found to be predictors of binge drinking [53]. The preliminary outcomes of the intervention proved effective in modifying intention for binge drinking in the desired direction. In light of the success of targeted strategies, the findings from this study may be used to implement interventions such as web-based smartphone applications to reduce problematic alcohol use in the indicated high risk groups. This avenue provides greater reach in university student groups and is therefore particularly effective to reduce the risk for high levels of alcohol consumption [54].

Limitations should be considered when interpreting the data because the parameters used in this study were solely survey-based or self-reported, which may give rise to misestimated information, i.e., PA/alcohol intake, to affect the outcomes of this study. However, previous studies have demonstrated the reliability and validity of self-reported surveys when conducting similar population studies [55]. Although our data were collected from three universities across several years, we did not find any significant change related to social or economic trends during these years that may influence alcohol behaviours in these universities. The generalisability of the current sample to the UK population, as documented by Universities UK [29], was reflected by similarity in gender, domicile and ethnicity percentages [30,31,32]. BMI percentages found in our study also aligned with percentages recognised across UK universities [56]. The response rate (94.1%) achieved was above the standard for avoiding nonresponse bias [57] however, the cross-sectional design prevents the derivation of any causal relationships. The reporting of screen-time sedentary behaviours may not reflect entire sedentary engagement. Alcohol intake behaviours may differ in various settings; the geographic distribution of the selected universities herein represents only the UK population or countries with similar alcohol cultures. Nevertheless, given the uncertainties in this field of research, this study attempts to disentangle the discrepancies of the factors which influence alcohol consumption in young adults using a large, recent and diverse university sample.

## 5. Conclusions

In summary, we have found that the levels of alcohol consumption in university young adults are influenced by gender, ethnicity, smoking, the levels of PA and recreational sedentary screen-time. Being a male, having a Caucasian ethnicity, smoking, and being physically active appear to be positively associated with high levels of alcohol consumption, whereas, engagement in greater amounts of recreational screen-based activities are negatively associated with the levels of alcohol consumption in males. The information from our study may aid policy makers in developing alcohol awareness initiatives and guide the promotion of conscious drinking particularly in the myriad of young adults who are at risk of drinking alcohol beyond the limit. This could lower the risk of alcohol-related harm including liver disorders, cancers, cardiometabolic diseases and premature mortality while potentially reducing related harm to families and society across the UK. Providing active gaming facilities in social clubs and bars and promoting non-sport team associated PA, particularly to males, on the university campus may help to divert the attention of young alcohol consumers and to reduce the frequency and the amount of binge drinking.

## Figures and Tables

**Table 1:** Demographic information and lifestyle behaviours among university students.

Variable	Males		Females		Total		*p*-Value
Gender	627		813		1440		<0.01
Age (years), mean (SD)	19.6 (2.17)		20.2 (3.03)		19.9 (2.73)		<0.01
BMI (kg/m^2^), mean (SD)	22.6 (4.95)		21.2 (5.24)		21.8 (5.16)		<0.01
	***n***	**%**	***n***	**%**	***n***	**%**	
Underweight (<18.5)	36	5.7	67	8.2	103	7.2	0.61
Normal (18.5–24.9)	437	69.7	636	78.2	1073	74.5	<0.01
Overweight/Obese (≥25/≥30)	154	24.6	110	13.5	264	18.3	0.19
**Ethnicity**							
Caucasian	328	52.3	455	56.0	783	54.4	0.17
Asian	113	18.0	158	19.4	271	18.8	0.49
Black African	47	7.5	41	5.0	88	6.1	0.05
Mixed	18	2.9	16	2.0	34	2.4	0.26
Other ethnic group	121	19.3	143	17.6	264	18.3	0.41
**Country of Residence**							
Europe	527	84.1	703	86.5	1230	85.4	0.19
Outside Europe	77	12.3	85	10.5	162	11.3	0.28
Not specified	23	3.7	25	3.1	48	3.3	
**Smoking Status**							
Non-smoker	545	86.9	745	91.6	1290	89.6	<0.01
Smoker	82	13.1	68	8.4	150	10.4	
**Physical Activity (h/week)**							
0	75	12.0	123	15.1	198	13.8	0.08
<2.5	212	33.8	301	37.0	513	35.6	0.21
2.5–8	277	44.2	346	42.6	623	43.3	0.54
>8	63	10.0	43	5.3	106	7.4	<0.01
**Sleeping (h/day)**							
<7	323	51.5	450	55.4	773	53.7	0.15
≥7	282	45.0	350	43.1	632	43.9	
Not specified	22	3.5	13	1.6	35	2.4	
**Recreational Sedentary Screen-Time (h/day)**							
<4	256	40.8	325	40.0	581	40.3	0.74
≥4	371	59.2	488	60.0	859	59.7	
**Perception of Physical Fitness**							
Unfit	295	47.0	487	59.9	782	54.3	<0.01
Fit	332	53.0	326	40.1	658	45.7	

**Table 2:** Adjusted odds ratios as effect measures of the influence on alcohol consumption of included lifestyle predictors separated by gender.

Assessments	Males (*n* = 627, 43.5%)			Females (*n =* 813, 56.5%)		
BMI (kg/m^2^)	*n* (%)	OR (CI)	*p*-value	*n* (%)	OR (CI)	*p*-value
Normal (18.5–24.9) *	437 (69.7)			636 (78.2)		
Underweight (<18.5)	36 (5.7)	1.49 (0.57–3.88)	0.41	67 (8.2)	0.86 (0.51–1.46)	0.86
Overweight/Obese (≥25/≥30)	154 (24.6)	1.10 (0.68–1.78)	0.70	110 (13.5)	1.3 (0.82–2.12)	0.25
**Ethnicity**						
Caucasian	328 (52.3)	4.55 (2.63–7.69)	<0.01	455 (56.0)	3.03 (2.04–4.48)	<0.01
Other ethnic group *	299 (47.7)			358 (44.0)		
**Smoking Status**						
Non-smoker *	545 (86.9)	3.5 (1.71–7.17)	<0.01	745 (91.6)	2.76 (1.50–5.08)	<0.01
Smoker	82 (13.1)			68 (8.4)		
**Physical Activity Levels (h/week)**						
0 *	75 (12.0)			123 (15.1)		
<2.5	212 (33.8)	2.46 (1.13–5.32)	0.02	301 (37.0)	1.25 (0.75–2.07)	0.04
2.5–8	277 (44.2)	3.76 (1.71–8.28)	<0.01	346 (42.6)	1.04 (0.62–1.74)	0.88
>8	63 (10.0)	5.47 (2.12–10.11)	<0.01	43 (5.3)	0.72 (0.33–1.58)	0.41
**Sleep Duration (h/day)**						
<7 *	323 (51.5)	0.77 (0.52–1.14)	0.19	450 (55.4)	0.86 (0.64–1.17)	0.33
≥7	282 (45.0)			350 (43.1)		
**Recreational Sedentary Screen-Time (h/day)**						
<4 *	256 (40.8)	0.31 (0.12–0.86)	0.03	325 (40.0)	0.63 (0.32–1.24)	0.18
≥4	371 (59.2)			488 (60.0)		
**Perception of Physical Fitness**						
Unfit *	295 (47.0)	0.77 (0.50–1.19)	0.24	487 (59.9)	1.22 (0.88–1.69)	0.24
Fit	332 (53.0)			326 (40.1)		

A generalised linear model with a multinomial distribution for an ordered categorical outcome (“alcohol consumption”) was used to yield adjusted OR (odds ratios). The OR serve as an effect measure of the influence of alcohol consumption on other lifestyle components in male and female students. BMI: body-mass index; CI: 95% confidence interval. * OR and CI of all sub-groups were computed using the marked reference group.

## Supplementary Materials

The following are available online at https://www.mdpi.com/1660-4601/17/17/6282/s1, Table S1: Statistical analysis of the levels of alcohol consumption between Caucasian and other ethnic groups by gender.
