# Supplementary material for: Exploration of Alcohol Consumption Behaviours and Health-Related Influencing Factors of Young Adults in the UK
Source: Int J Environ Res Public Health. 2020 Aug 28;17(17):6282. doi: 10.3390/ijerph17176282 (PMC7503755; doi:10.3390/ijerph17176282)
Supplement: Supplementary file 1 [file ijerph-17-06282-s001.pdf]

Supplemental Table 1. Statistical analysis of the levels of alcohol consumption between Caucasian and other ethnic groups by gender

Variable	Males (n = 627, 43.5%)			Females (n= 813, 56.5%)		
**Ethnicity**	***n*** **(%)**	**OR (CI)**	***p*-value**	***n*** **(%)**	**OR (CI)**	***p*-value**
Caucasian*	328 (52.3)			455 (5.60)		
Asian	113 (18.0)	0.22 (0.13-0.37)	0.00	158 (19.4)	0.12 (0.08-1.19)	0.00
Black African	47 (7.5)	0.12 (0.06-0.26)	0.00	41 (5.0)	0.11 (0.05-0.23)	0.00
Mixed	18 (2.9)	0.08 (0.02-0.32)	0.00	16 (2.0)	0.45 (0.17-1.16)	0.09
Other	121 (19.3)	0.22 (0.13-0.38)	0.00	143 (17.6)	0.33 (0.22-0.49)	0.00

p-value assessing the overall effect of each sub-group within ethnicity by gender, as output by the statistical model on the multinomial outcome (“alcohol consumption”) which yielded adjusted OR. OR: Odds Ratio; CI: 95% confidence interval. *OR and CI of all sub-groups were computed using Caucasian as the reference group.
